# Novel Simian Foamy Virus infection of wild Indian rhesus macaque (Macaca mulatta)

**DOI:** 10.1186/1742-4690-8-S1-A215

**Published:** 2011-06-06

**Authors:** Jayashree S Nandi, Anil K Chhangani, Surendra M Mohnot

**Affiliations:** 1 Dept. of Microbiology and Immunology, Albert Einstein College of Medicine, Bronx, New York ,10461, USA; 2 Dept. of Zoology, Jai Narain Vyas (JNV) University, Jodhpur, India; 3 Primate Research Centre, Jodhpur, India

## 

Simian Foamy Viruses (SFVs) infect many non-human primates, (NHP) by species-specific strain of SFV. Infection in humans occurs through zoonosis form NHP in areas where hunting for bush meat is common, as in Africa. To investigate natural retroviral infection of wild Indian simians, blood samples were collected from forested regions of Rajasthan state. Using degenerate primers from the pol region of retroviral genome, 3 out of 24 (~12%) wild rhesus macaque (Macaca mulatta) gave positive signals. Direct sequencing of PCR products revealed novel SFV infection of wild Indian rhesus macaques, M1, M2 and M4. The sequences were aligned with known SFV sequences from various NHP species. The SFV sequences from pol (Integrase) region had the characteristic core domain and the conserved zn finger domains (His 2-Cys2). Pol sequences of SFV infecting wild Indian rhesus macaques were 8-10% divergent from available SFVmac sequences. Overall the novel SFV sequences were related to other SFV sequences from Genbank but formed a unique cluster in Neighbor Joining phylogenetic tree. Close man-monkey interaction has existed in India for centuries because of the religious connotation of simians, but bush meat consumption is not common except in some tribal populations from the northeastern regions. Future investigation with specific rather than degenerate primers will determine the actual prevalence of SFV infection in wild simian population from India. Similarly, analyses of blood samples from humans with history of bleeding monkey bite at temple and tourist sites will reveal if simian to human transmission of SFV exists in India.

